# Whole-genome sequencing of the first *Candida auris* clinical isolate from a patient with sepsis in Palestine

**DOI:** 10.1128/mra.01239-24

**Published:** 2025-03-31

**Authors:** Rasmi Abu-Helu, Faiza Tebbji, Ahmad Al Bishawi, Hanaa Baniodeh, Antony T. Vincent, Adnane Sellam

**Affiliations:** 1 Department of Medical Laboratory Sciences, Faculty of Health Professions, Al-Quds University 61328 , Jerusalem, Palestine; 2 Montreal Heart Institute/Institut de Cardiologie de Montréal, Université de Montréal, Montréal, Québec, Canada; 3 Department of Infectious Diseases, Ibn Sina Specialized Hospital, Jenin, Palestine; 4 Department of Animal Sciences, Université Laval 4440 , Québec City, Québec, Canada; 5 Department of Microbiology, Infectious Diseases and Immunology, Faculty of Medicine, Université de Montréal, Montréal, Québec, Canada; University of Guelph, Guelph, Canada

**Keywords:** *Candida auris*, antifungal resistance, complete genome sequence

## Abstract

An isolate of the multidrug-resistant yeast *Candida auris* (Pal1) was identified in the urine of a patient with sepsis and sequenced using both Oxford Nanopore and Illumina sequencing technologies. Phylogenetic analysis placed *C. auris* Pal1 in the geographical Clade I, with distinct SNPs linked to azole resistance (Erg11^Y132F^ and Mrr1^T647N^).

## ANNOUNCEMENT

*Candida auris* has emerged as a significant healthcare-associated pathogen due to its multidrug-resistant nature, and it is listed in the WHO critical priority group due to its serious global health threat. In the first prevalence study of *Candida* infections in Palestine, many isolates of *Candida* spp. were not identified at the species level (1). As many of these isolates were resistant to fluconazole, we decided to specifically screen for *C. auris. C. auris* Pal1 was isolated from the urine of a patient with severe sepsis admitted at Ibn Sina Hospital (Jenin, Palestine) using CHROMagar *Candida* medium (CHROMagar, Paris, France). Pal1 isolate was initially identified as *C. auris* by the VITEK2 Compact system. A single colony of this isolate was inoculated into YPD (1% Yeast extract, 2% Peptone, 2% Dextrose, and 50 µg/mL uridine) medium and incubated at 30°C overnight. The cells were then harvested by centrifugation, and genomic DNA was extracted using the YeaStar kit (Zymo Research). Genomic DNA was first subjected to Sanger sequencing of the ITS2 region (2) (PQ578863) that confirmed this isolate as a *C. auris* species. Whole-genome sequencing was performed at Plasmidsaurus (Eugene, OR, USA) using both Oxford Nanopore Technology (ONT) long-read and Illumina NextSeq2000 short-read sequencing technologies. The Nanopore library was prepared using v14 library prep chemistry without fragmentation or size selection and sequenced on the PromethION P24 platform (Oxford Nanopore Technologies), equipped with R.10.4.1 flow cells. The Illumina library was made using the SeqWell ExpressPlex 96 library prep kit and sequenced on an Illumina NextSeq2000 (2 × 150 bp).

Illumina sequencing reads were filtered with Fastp version 0.23.2 (3) and those from Nanopore were filtered using Filtlong version 0.2.1 (https://github.com/rrwick/Filtlong) by keeping the best 90% of reads above 1,000 bp. Read quality was verified with FastQC version 0.12.1 (https://www.bioinformatics.babraham.ac.uk/projects/fastqc/). After filtration, 2.25 million Illumina reads were retained, totaling 354 Mb (96% > Q20, 85% > Q30). For Nanopore reads, 286,335 reads (*N*_50_ ~11 kb) were retained, totaling 2.4 Gb. Nanopore reads were assembled with Flye version 2.9.5-b1801 (4) at an average coverage of 200×. The assembly was then polished with Polypolish version 0.6.0 (5). The final assembly resulted in eight contigs, corresponding to the seven chromosomes and the mitochondrial genome. Annotation was performed with Funannotate version 1.8.17 (https://github.com/nextgenusfs/funannotate/) using default parameters and the *Candida albicans* SC5314 model (http://www.candidagenome.org), which is the closest available species.

**Fig 1:**
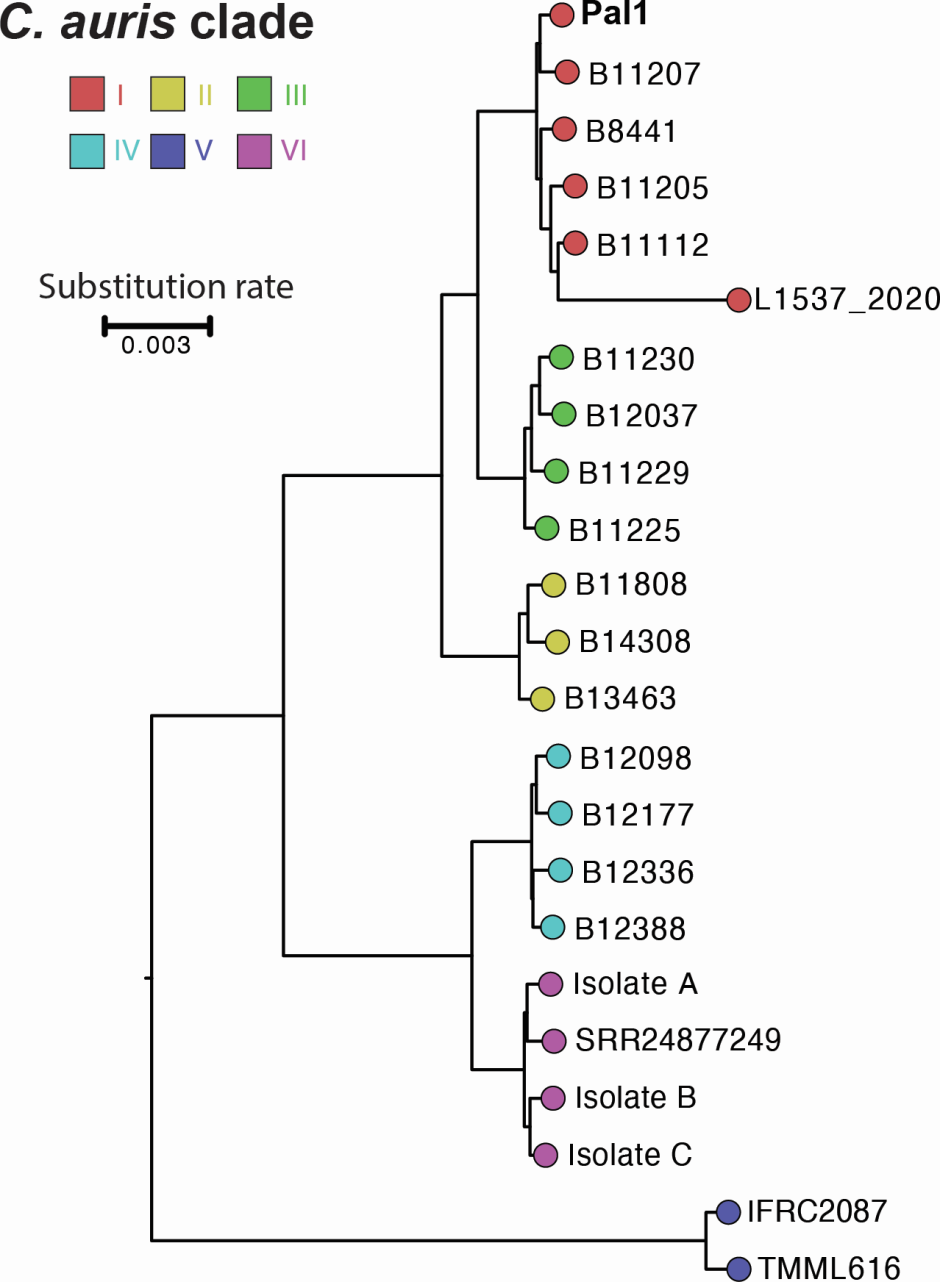
Average nucleotide identity (ANI) analysis showing genetic relatedness of the *C. auris* Pal1 isolate with the genomes of 22 *C*. *auris* isolates representative of the six geographical clades (B11205: SRX1939460, B11207: SRX1939463, L1537_2020: SRX10155110, B8441: SRX7522312, B11112: SRX1939497, B11808: SRX7155656, B14308: SRX7155771, B13463: SRX7155759, B11229: SRX1939486, B11230: SRX1939487, B11225: SRX1939487, B12037: SRX7155666, B12388: SRX4745454, B12177: SRX7155717, B12336: SRX4060912, B12098: SRX7155671, IFRC2087: SRX5786024, TMML616: SRX14462689, SRR24877249: SRX20641053, Isolate A: SRX21188005, Isolate B: SRX21188006, and Isolate C: SRX21188007). The scale bar indicates the mean number of nucleotide substitutions per site.

*C. auris* Pal1 relatedness to different isolates that are representative of the six known geographical clades (6) was evaluated using the average nucleotide identity method (7). This analysis showed that Pal1 is closely related to isolate B11207 (8) that belongs to the geographical Clade I (9) (Fig. 1). Pal1 isolate harbors different SNPs associated with azole resistance, including Erg11^Y132F^ and Mrr1^T647N^.

## Data Availability

The genome sequences of the current study are available in GenBank under BioProject and BioSample accession number PRJNA1185736 and SAMN44720146, respectively. The raw reads are available in the Sequence Read Archive (SRA) under accession number SRS23215131. The internal transcribed spacer 2 (*ITS2*) sequence of *C. auris* Pal1 was determined by Sanger sequencing and deposited in GenBank under the accession number PQ578863.
